# Effects of a Partially Perforated Flooring System on Ammonia Emissions in Broiler Housing—Conflict of Objectives between Animal Welfare and Environment?

**DOI:** 10.3390/ani11030707

**Published:** 2021-03-05

**Authors:** Carolin Adler, Alexander J. Schmithausen, Manfred Trimborn, Sophia Heitmann, Birgit Spindler, Inga Tiemann, Nicole Kemper, Wolfgang Büscher

**Affiliations:** 1 Institute of Agricultural Engineering, University of Bonn, 53115 Bonn, Germany; m.trimborn@uni-bonn.de (M.T.); inga.tiemann@uni-bonn.de (I.T.); buescher@uni-bonn.de (W.B.); 2 Corteva Agriscience, Riedenburger Straße 7, 81677 München, Germany; alexander.schmithausen@corteva.com; 3 Institute of Animal Hygiene, Animal Welfare and Farm Animal Behaviour, University of Veterinary Medicine Hannover, Foundation, 30559 Hannover, Germany; sophia.heitmann@tiho-hannover.de (S.H.); birgit.spindler@tiho-hannover.de (B.S.); nicole.kemper@tiho-hannover.de (N.K.); 4 Institute of Animal Science, University of Bonn, 53115 Bonn, Germany

**Keywords:** broiler production, alternative flooring, ammonia emissions, animal welfare, environmental impact

## Abstract

**Simple Summary:**

Previous studies have shown positive effects of a partially perforated flooring system on animal welfare in broiler housing. Towards the end of the fattening periods, the present study showed a higher ammonia emission rate (NH_3_ ER) for a partially perforated flooring system compared with a littered control barn. Nevertheless, the measured NH_3_ concentrations were below 20 ppm, except during a mechanical litter treatment in the winter fattening period. Furthermore, the system offers the possibility of applying practical solutions that were not feasible before. By using underfloor air extraction, manure belts, or acidification systems underneath the elevated perforated area, NH_3_ concentrations and the resulting NH_3_ ER could be reduced. Thus, with some optimization, the partially perforated flooring system could be used to contribute to an increase in animal welfare and environmental protection at the same time.

**Abstract:**

A partially (50%) perforated flooring system showed positive effects on health- and behavior-based welfare indicators without affecting production performance. Ammonia (NH_3_) is the most common air pollutant in poultry production, with effects on animal welfare and the environment. The objectives of animal welfare and environmental protection are often incompatible. Therefore, this study addresses the question of how a partially perforated flooring system affects NH_3_ emissions. According to German regulations, three fattening periods were carried out with 500 Ross 308 broilers per barn (final stocking density: 39 kg m^−2^). The experimental barn was equipped with an elevated perforated area in the supply section, accessible by perforated ramps. The remaining area in the experimental barn and the control barn were equipped with wood shavings (600 g m^−2^). Besides the different floor types, management was identical. Air temperature (Temp), relative air humidity (RH), NH_3_ concentration, and ventilation rate (VR) were measured continuously. Furthermore, dry matter (DM) content, pH, and litter quality were assessed. Towards the end of the fattening periods, the NH_3_ emission rate (ER) of the partially perforated flooring system was higher compared with that of the littered control barn (all *p* < 0.001). This effect is mainly caused by the higher NH_3_ concentrations, which are promoted by the lack of compaction underneath the elevated perforated area and the increase in pH value under aerobic conditions. Nevertheless, the partially perforated flooring system offers different approaches for NH_3_ reduction that were previously not feasible, potentially contributing equally to animal welfare and environmental protection.

## 1. Introduction

In Germany, broilers are conventionally kept on concrete floors equipped with organic bedding materials [1]. From day 14, about 80% of the litter’s dry matter (DM) consists of excrements and feed residues [2]. If the drying conditions are unfavorable, footpad dermatitis, hock burn, and plumage contamination occur [3,4,5]. As the abovementioned aspects lead to pain and secondary diseases, the incidence and severity are used as animal-based indicators to record animal welfare [6,7,8]. In addition, the litter provides a potential reservoir for antibiotic-resistant bacteria [9] that can be transmitted to humans via the food chain [10,11]. Two studies were carried out in the past comparing an innovative partially (50%) perforated flooring system with a littered flooring system [12,13]. One feature of the partially perforated flooring system is an elevated perforated area in the section of feed and water supply, accessible by perforated ramps. Next to the elevated perforated area, littered areas are available. The system provides access to two different floor types at different height levels and promotes the animals’ natural behavior such as perching and resting on elevated levels [14,15] or pecking, scratching, and dustbathing in contact with litter [16,17]. At the same time, animals are separated from at least 50% of the litter, which contains excrements, moisture, and bacteria. Adler et al. [12] examined the effect of the partially perforated flooring system on animal-based welfare indicators and production performance. The creation of different functional areas at different heights enriched the husbandry environment, increased the environmental complexity, and reduced animals’ general fear response, as confirmed by [18,19]. Furthermore, the separation of the animals from at least 50% of the litter had a positive influence on foot pad dermatitis and hock burn. Production performance was not affected by the floor type [12]. Heitmann et al. [13] studied the effect of the partially perforated flooring system on the occurrence of bacteria. A tendency was shown for a higher content of *Escherichia coli* (*E. coli*) in the supply area of the partially perforated system compared with the supply area of the littered flooring system. Owing to the elevated perforated area, the animals did not come into contact with the material containing *E. coli* underneath the perforated floor. Regarding the total bacteria count, a tendency for lower contents in the littered side areas of the partially perforated flooring system was found compared with the littered control barn. In summary, the partially perforated flooring system has a positive effect on health- and behavior-based welfare indicators without a reduction in production performance [12].

Due to the conflict of objectives between animal welfare and the environment, it is also important to consider the influence of the partially perforated flooring system on environmental aspects [20]. Ammonia (NH_3_) is the most common air pollutant emitted by poultry production [21]. Several studies revealed that NH_3_ has negative effects on human and animal health as well as the environment. NH_3_ inside the barn is known to irritate the mucous membranes and damage the respiratory tract of humans and animals [22,23,24]. Negative effects on broilers’ production performance as a result of NH_3_ concentrations above 30 ppm were also observed [25,26]. Furthermore, NH_3_ released in the air from poultry houses is able to contribute to the production of acid rain [27] and therefore nitrogen (N) deposition in the ecosystem [28]. Potential consequences of N deposition are eutrophication, acidification, less biodiversity, and nitrification of groundwater [29].

Several studies have been carried out on different flooring systems with regard to NH_3_. For example, Boggia et al. [30] showed a reduction in the NH_3_ concentration using a no-litter flooring system for broilers compared with conventional litter flooring. Almeida et al. [31] used a totally (100%) perforated flooring system and showed a reduction in the NH_3_ concentration, if manure was continuously removed during the fattening period. In the case of manure storage underneath a totally (100%) perforated flooring system over several fattening periods, the NH_3_ concentrations and the resulting NH_3_ emissions were higher compared with litter flooring [32].

It is known that the partially perforated flooring system has positive effects on animal-based welfare indicators [12]. So far it is not known how the partially perforated flooring system affects NH_3_ emissions and whether harmful NH_3_ concentrations are to be expected. Therefore, the aim of this study was to investigate the effect of the partially perforated flooring system on NH_3_ emissions compared with a littered system.

## 2. Materials and Methods

### 2.1. Animals and Housing

This case-control study was carried out at the Educational and Research Center Frankenforst of the Faculty of Agriculture, University of Bonn (Königswinter, Germany; 55°42′55 N and 7°12′26 E). The experiments were performed in accordance with German regulations and approved by the relevant authority (Landesamt für Natur-, Umwelt- und Verbraucherschutz Nordrhein-Westfalen, Recklinghausen; 81.02.04.2018.A057). A total of three fattening periods were carried out in three different seasons from August 2018 to June 2019. Due to specifications of the slaughterhouse, each fattening period lasted 31 to 32 days. The study was carried out according to German regulations by housing 500 Ross 308 broilers per barn and fattening period to achieve a final stocking density of 39 kg m^−2^. Two identical barns were used to fulfill the conditions of a case-control study (experimental vs. control barn). Both barns were automatically ventilated by negative pressure ventilation, regulated identically via climate computers (PL-9400, Stienen Bedrijfselektronica B.V., RT Nederweert, The Netherlands). More information on management, feeding, lighting program, and vaccination can be found in the previous study by Adler et al. [12].

### 2.2. Floor Design and Litter Management

**Figure 1:**
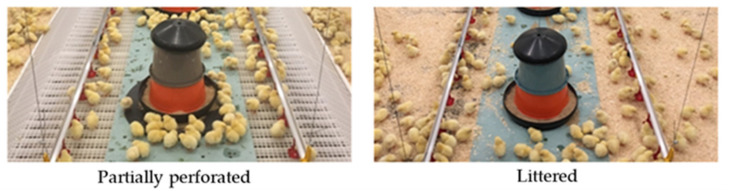
Illustration of the partially perforated flooring system, with an elevated perforated area in the section of feed and water supply, and the littered flooring system.

Figure 1 illustrates the two different flooring systems. The experimental barn was equipped with an elevated perforated floor in the area of feed and water supply, accessible by perforated ramps. Conventional wood shavings (600 g m^−2^) were used in the concrete floor areas next to the feed and water supply. The control barn was completely equipped with wood shavings (600 g m^−2^).

After each fattening period, both barns were cleaned, disinfected, and provided with new wood shavings. During the winter fattening period, the litter was additionally mechanically treated with a rake on day 11 to ensure an even distribution in all areas of the barn, especially in the area near the ramps.

### 2.3. Indoor Environmental Factors

**Figure 2:**
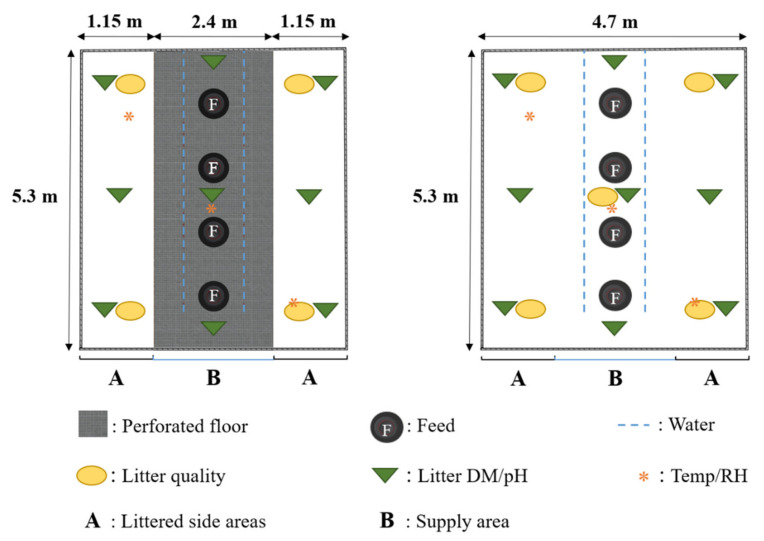
Floor plan view of the partially perforated flooring system, with an elevated perforated area in the section of feed and water supply, and the littered flooring system, including measurement positions for litter quality, litter dry matter (DM), litter pH (pH), air temperature (Temp), and relative air humidity (RH).

Inside the barn, air temperature (Temp) and relative air humidity (RH) were measured every three minutes using data loggers (Tinytag Plus 2—TGP-4500 loggers, Gemini Data Loggers Ltd., Chichester, West Sussex, UK). A total of three data loggers per barn were placed in the same positions and at a height of 55 cm (Figure 2). Data regarding Temp and RH in the environment outside the barns were provided every 10 min by the nearby weather station at a height of 2.0 m (Königswinter, Germany; 50°42′54 N and 7°12′31 E).

### 2.4. Ammonia Concentration

The NH_3_ concentrations were measured continuously via photoacoustic infrared spectroscopy using a Photoacoustic Gas Monitor INNOVA 1412 in combination with a Multipoint Sampler INNOVA 1309 (LumaSense Technologies SA, Ballerup, Dennmark). The measurement setup was based on studies carried out by Schmithausen et al. [33,34]. Additionally, the gas monitor was calibrated by the manufacturer and regularly checked in the measuring laboratory between the fattening periods. In total, three sampling points were installed with filter orifices to protect the technique from dust. Two sample points were installed in the exhaust chimneys of the barns. A third sampling point was installed outside the barns to measure the background concentration. The air of each sampling point was collected continuously by vacuum pumps (ME 2C, Vacuubrand GmbH + Co. KG, Wertheim, Germany) through polytetrafluoroethylene tubes in three separate sample bottles (600 mL). The tubes were equipped with heating cables (A. Rak Wärmetechnik GmbH, Frankfurt am Main, Germany) to avoid water condensation in the tubes with temperature decrease. Due to the continuously flushed sample bottles and tube system, there was always actual sample air available. The tubes between the sample bottles and the Multipoint Sampler were as short as possible (<0.5 m). This measurement setup is useful to ensure a small distance from the sampling point until the analysis. One measuring cycle lasted three minutes with a measuring time of 60 s per sampling point.

### 2.5. Ventilation Rate

The ventilation rate (VR) in both exhaust chimneys was estimated using ProVent measurement fans (Reventa GmbH, Horstmar, Germany) of the same diameter as the exhaust chimney. The measuring fans were calibrated in a wind tunnel by the manufacturer. There was a calming distance of 2.0 m between the air outlet and the measuring fan to fulfill laminar air flow conditions. These conditions are useful to increase the measurement accuracy of the measurement fans. The data were recorded every minute by Almemo 2590 data loggers (Ahlborn Mess- und Regelungstechnik GmbH, Holzkirchen, Germany).

### 2.6. Litter Analysis

At the end of each fattening period on day 31 to 32, representative litter samples were taken to determine the litter dry matter (DM). In the spring fattening period, further litter samples were taken on days 7, 14, 21, and 28. Litter samples were taken in nine different positions: a total of six positions in the littered side areas and three positions in the supply area of each barn (Figure 2). In each position, a sample of the entire depth of the litter was taken. The litter samples were weighed before and after being oven-dried at 105 °C for 24 h.

In addition, the litter samples of the spring fattening period were analyzed regarding the pH value. A total amount of 20 g of each litter sample was mixed with 300 g deionized water. The samples were then shaken with an overhead shaker (Reax 20 overhead shaker, Heidolph Instruments GmbH & Co. KG, Schwabach, Germany) for a total time of one hour (30 turns m^−1^). After shaking, the pH was measured using a pH electrode (InLab Max Pro-ISM electrode, Mettler Toledo, OH, USA). The pH electrode was calibrated using a buffer solution for pH 4 and 7.

### 2.7. Litter Quality

**Figure 3:**
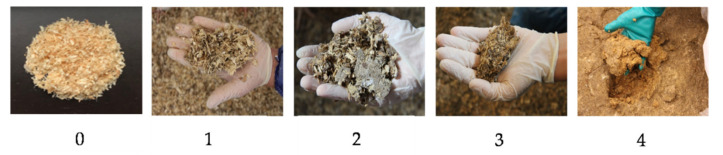
Illustration of the scores used during the litter quality evaluation.

In all three fattening periods, litter quality was assessed on days 7, 14, 21, and 28 using the scoring system developed by Welfare Quality^®^ [35]. Figure 2 shows the positions where litter quality was evaluated. A total of five litter samples were taken in the littered control barn, with four samples in the littered side areas and one sample in the supply area. In the experimental barn, four litter samples were taken in the littered side areas. No litter quality assessment was performed underneath the perforated area in the supply area of the experimental barn. Underneath the perforated area, mainly excrements are stored, which cannot be defined and evaluated as litter. A scoring system from 0 to 4 was used to evaluate the litter quality [35]. Figure 3 illustrates the images of the different scores. Score 0 was equal to “completely dry and flaky, that is, moves easily with the foot”; score 1 was equal to “dry but not easy to move with foot”; score 2 was equal to “leaves imprint of foot and will form a ball if compacted, but ball does not stay together well”; score 3 was equal to “sticks to boots and sticks readily in a ball if compacted”; and score 4 was equal to “sticks to boots once the cap or compacted crust is broken.”

### 2.8. Data Processing and Statistical Analysis

For statistical analysis, SPSS^®®^ Statistics 25 (IBM Corporation, Armonk, NY, USA) was used. Graphical presentation was done with SigmaPlot 14.0 (Systat Software Inc., Chicago, IL, USA). The daily emission rate (ER) was calculated using the average hourly NH_3_ concentrations (*n =* 20 values per hour and sample point) and the average hourly ventilation rates (*n =* 60 values per hour and barn) using the following equation:

ER = (((C_inside_ − C_outside_) × VR)/N) × 24 (1)

where:

ER = emission rate (g d^−1^ bird^−1^)
C_inside_ = inside ammonia concentration (g m^−3^)
C_outside_ = outside ammonia concentration (g m^−3^)
VR = ventilation rate (m^3^ h^−1^)
N = actual number of birds per day

For each fattening period, the hourly values for NH_3_ ER, NH_3_ concentration, VR, air Temp, and RH were divided into three sections according to the feeding program: start (day 0 to 6), middle (day 7 to 27), and end of the fattening period (day 28 to 31 or 32). Data were analyzed using general linear models (GLMs). For litter quality, the link function was Poisson distributed, otherwise it was linear distributed. In the first step, univariate GLMs were used to select the significant main effects with NH_3_ ER, NH_3_ concentration, VR, air Temp, RH, litter quality, DM, and pH as response variables. Significant main effects were then analyzed by a multifactorial GLM. After backward selection, the final GLMs were interpreted with interaction terms (Fattening period × Section × Floor type, Area × Fattening period, or Area × Day). The *p*-values were corrected by Bonferroni. Differences of *p* ≤ 0.05 were considered statistically significant, and differences of 0.05 ≤ *p* ≤ 0.10 were considered a tendency.

## 3. Results

### 3.1. Indoor Environmental Factors

**Figure 4:**
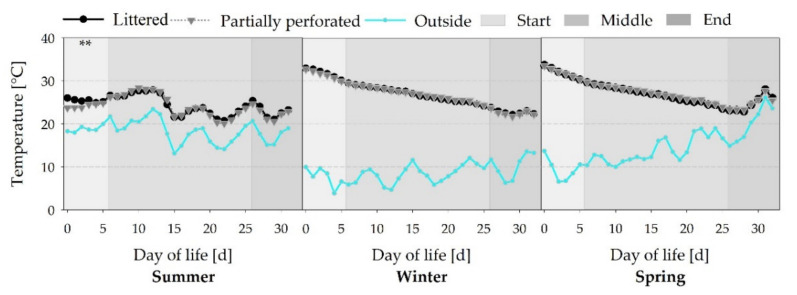
Average daily air temperature inside the broiler houses with two different floor types, and the outside environment air temperature measured over three different fattening periods (*n =* 144 values per day and barn). Significant differences between both floor types within the three different sections (start, middle, end of the fattening period) are marked by asterisks: ** *p* < 0.01.

Figure 4 shows typical Temp curves with a decreasing Temp towards the end of the fattening periods (all *p* < 0.001). During the summer fattening period, no additional heating sources were used. Therefore, the barn Temp of the summer fattening period was more oriented to the outside Temp. The initial Temp differences between both floor types in the summer fattening period are due to the settings of the climate computer (*p* < 0.01).

**Figure 5:**
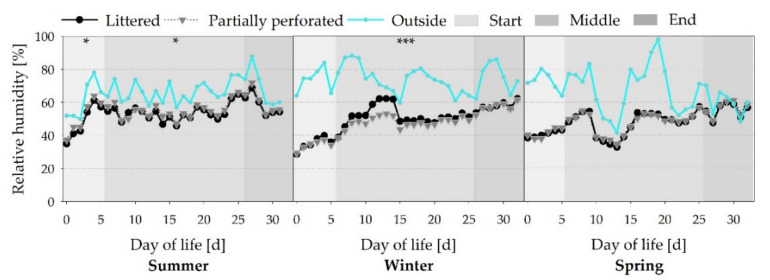
Average daily relative air humidity inside the broiler houses with two different floor types, and the outside environment RH measured over three different fattening periods (*n =* 144 values per day and barn). Significant differences between both floor types within the three different sections (start, middle, end of the fattening period) are marked by asterisks: * *p* ≤ 0.05, *** *p* < 0.001.

An increase in RH from the start to the end of the three fattening periods is presented in Figure 5 (all *p* < 0.001). Analogous to the Temp, the summer fattening period is oriented to the outside RH. Differences in RH between the flooring systems in the start and middle sections of the summer fattening period are due to the settings of the climate computer (all *p* < 0.05). On day 11 of the winter fattening period, the litter was mechanically treated, resulting in an RH peak. At this time, the RH was higher for the littered control barn compared with the partially perforated flooring system (*p* < 0.001).

### 3.2. Ammonia Concentration

**Figure 6:**
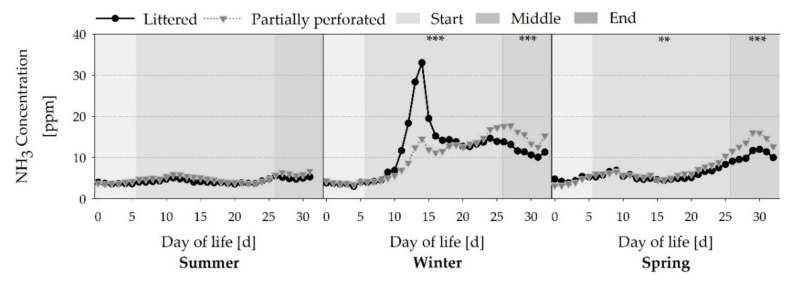
Average daily ammonia (NH_3_) concentrations from broiler houses with two different floor types measured over three different fattening periods (*n =* 480 values per day and barn). Significant differences between both floor types within the three different sections (start, middle, end of the fattening period) are marked by asterisks: ** *p* < 0.01, *** *p* < 0.001.

Figure 6 shows a typical increase in NH_3_ concentration towards the end of the fattening periods (all *p* < 0.01). Higher ventilation rates in the summer fattening period reflect lower NH_3_ concentrations (all *p* < 0.001). Owing to the mechanical litter treatment on day 11 of the winter fattening period, an NH_3_ concentration peak occurred. This peak was higher in the littered control barn than in the barn with the partially perforated flooring system (*p* < 0.001). Towards the end of the winter and spring fattening periods, NH_3_ concentrations of the partially perforated flooring system were higher compared with the littered control barn (all *p* < 0.01).

### 3.3. Ventilation Rate

**Figure 7:**
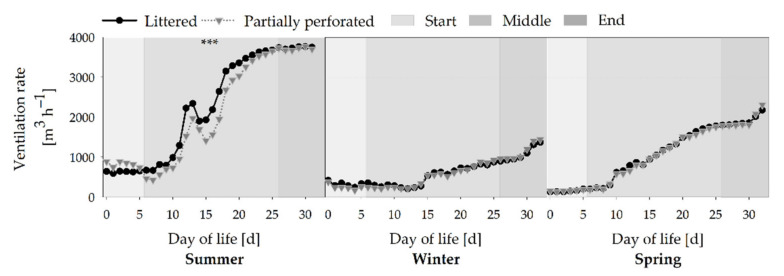
Average daily ventilation rate from broiler houses with two different floor types measured over three different fattening periods (*n =* 1.440 values per day and barn). Significant differences between both floor types within the three different sections (start, middle, end of the fattening period) are marked by asterisks: *** *p* < 0.001.

A typical course with an increasing VR towards the end of the fattening periods is shown in Figure 7 (all *p* < 0.001). Depending on the seasons, the highest VR is shown in the summer compared with the winter and spring fattening periods (all *p* < 0.001). Differences between both floor types in the middle section of the summer fattening period are due to the settings of the climate computer (*p* < 0.001). The VR is a preset parameter of the climate computer, resulting in parallel VR curves.

### 3.4. Ammonia Emission Rate

**Figure 8:**
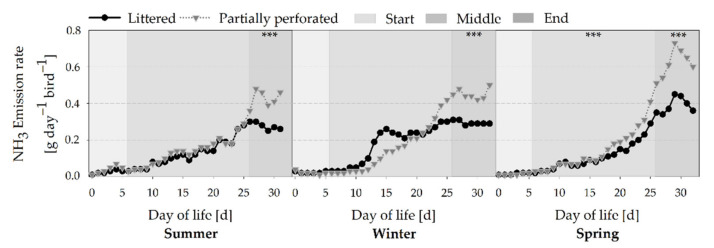
Average daily ammonia (NH_3_) emission rate from broiler houses with two different floor types measured over three different fattening periods (*n =* 24 values per day and barn). Significant differences between both floor types within the three different sections (start, middle, end of the fattening period) are marked by asterisks: *** *p* < 0.001.

Figure 8 shows a common NH_3_ ER increase over the time for all fattening periods (all *p* < 0.001). Overall, the average NH_3_ ER of the three fattening periods was lower for the littered control barn (0.15 ± 0.12 g day^−1^ bird^−1^) compared with the partially perforated flooring system (0.19 ± 0.19 g day^−1^ bird^−1^). These differences were especially observed towards the end of the fattening periods, with a lower NH_3_ ER for the littered control barn than for the partially perforated flooring system (all *p* < 0.001). Drifting apart of the NH_3_ ER between the two flooring systems began on days 26, 22, and 18 for the summer, winter, and spring fattening periods, respectively. Furthermore, a stagnation of the NH_3_ ER could be observed in the end section of the littered control barn in the summer and winter fattening periods.

### 3.5. Litter Analysis

**Figure 9:**
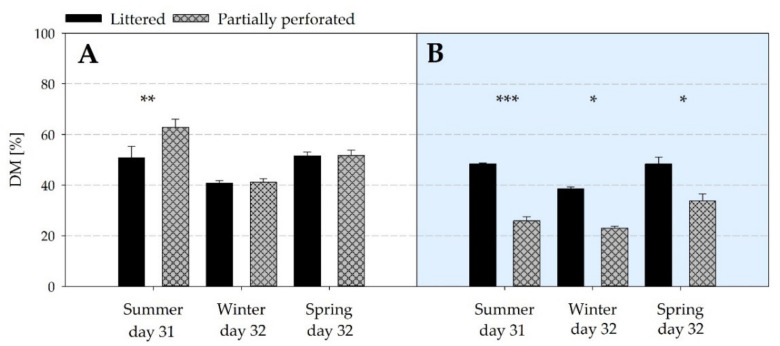
Average dry matter (DM) contents of litter samples from (**A**) the littered side areas and (**B**) the supply area from broiler houses with two different floor types at the end of each of the three different fattening periods (*n =* 6 values per fattening period and barn in area (**A**); *n =* 3 values per fattening period and barn in area (**B**)). Significant differences are marked by asterisks: * *p* ≤ 0.05, ** *p* < 0.01, *** *p* < 0.001.

The average litter DM at the end of the fattening periods is shown in Figure 9. At the end of the summer fattening period, litter DM was higher in the barn with the partially perforated flooring system compared with litter flooring (*p* = 0.004). In the supply area, litter DM was always lower for the partially perforated flooring system (all *p* < 0.032).

**Figure 10:**
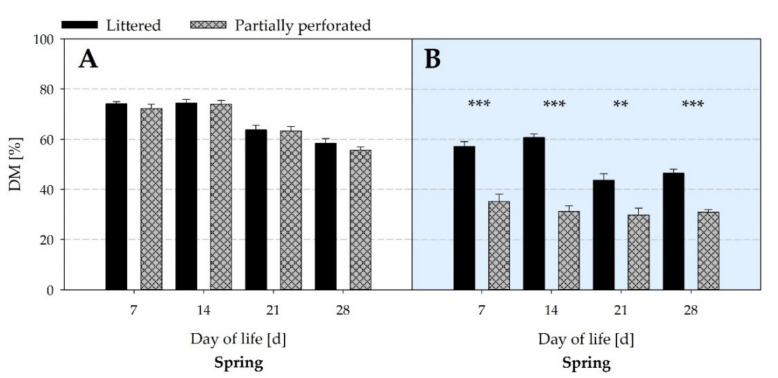
Average dry matter (DM) contents of litter samples from (**A**) the littered side areas and (**B**) the supply area (*n =* 6 values per day and barn in area (**A**); *n =* 3 values per day and barn in area (**B**)) from broiler houses with two different floor types measured over the spring fattening period. Significant differences are marked by asterisks: ** *p* < 0.01, *** *p* < 0.001.

The effect of a lower litter DM in the supply area of the experimental barn compared with the control barn is also reflected in Figure 10 over the whole spring fattening period (all *p* ≤ 0.01).

**Figure 11:**
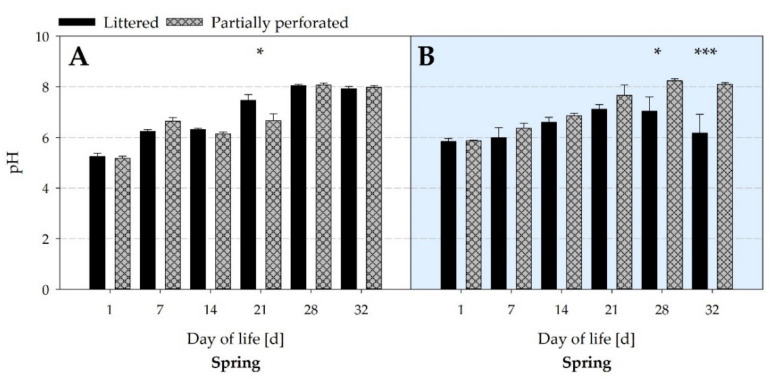
Average pH values of litter samples from (**A**) the littered side areas and (**B**) the supply area from broiler houses with two different floor types measured over the spring fattening period (*n =* 6 values per day and barn in area (**A**); *n =* 3 values per day and barn in area (**B**)). Significant differences are marked by asterisks: * *p* ≤ 0.05, *** *p* < 0.001.

Figure 11 presents the average daily litter pH values of the spring fattening period. In the littered side areas, litter pH increased from day 1 to 32 for both floor types (all *p* < 0.001). On day 21, litter pH in the littered side areas was higher in the control barn compared with the partially perforated flooring system (*p* = 0.032). In the supply area of the control barn, litter pH increased from day 14 to 28 and dropped back to the starting level by day 32 (all *p* < 0.015). By contrast, there was a continuous increase in litter pH from day 14 in the supply area of the barn with the partially perforated flooring system (all *p* < 0.001). From day 28, litter pH in the supply area was higher in the barn with the partially perforated flooring system compared with litter flooring (all *p* < 0.015).

### 3.6. Litter Quality

**Figure 12:**
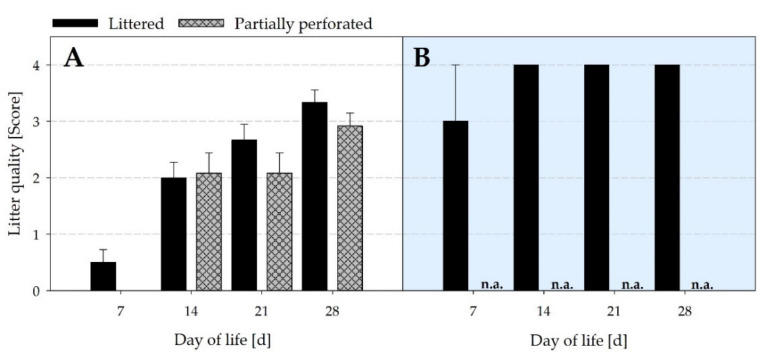
Mean scores of litter quality from (**A**) the littered side areas (*n =* 12 values per day and barn) and (**B**) the supply area (*n =* 3 values per day and barn) from broiler houses with two different floor types. n.a. = not assessed (no litter quality assessment underneath the perforated area in the supply area of the experimental barn).

Figure 12 presents a decrease in the litter quality of the littered side areas from the start to the end of the fattening periods (*p* < 0.001). For the littered control barn, litter quality was higher in the littered side areas compared with the supply area (*p* = 0.002). In the supply area of the littered control barn, the highest litter quality score was achieved from day 14 with a fully compacted litter surface. This score was never found in the littered side areas for both floor types. No litter quality assessment was carried out in the supply area of the experimental barn, as the accumulated excrements underneath the perforated area cannot be scored as litter.

## 4. Discussion

A multifactorial evaluation of an innovative flooring system in broiler production is important to ensure the compatibility of animal welfare and the environment. Previous studies have shown positive effects of a partially perforated flooring system on animal-based welfare indicators, without affecting production performance or the occurrence of bacteria [12,13]. Positive effects of elevated perforated areas on health- and behavior-based welfare indicators are confirmed by numerous studies [18,19,36,37,38,39]. Conflicting information is available regarding the environmental impact of a totally perforated flooring system, with a focus on NH_3_ concentration and ER [31,32]. No results are published on the effect of a partially perforated flooring system on NH_3_ concentration and ER.

By measuring the NH_3_ concentrations and VR, the NH_3_ ER was calculated. Results showed that the NH_3_ ER of the partially perforated flooring system was higher compared with the littered control barn towards the end of the fattening periods. Since no differences occurred in the VR between both flooring systems at that time, the higher NH_3_ ER is explained by the higher NH_3_ concentrations. As reported by Groot Koerkamp [40], NH_3_ is mainly generated from the decomposition of uric acid and undigested protein excreted by the broilers. NH_3_ volatilization depends on several factors such as VR, indoor Temp, and RH, as well as litter characteristics such as DM and pH [41]. In the current study, no differences in Temp and RH were measured towards the end of the fattening periods. Litter DM in the supply area was higher in the control compared with the experimental barn. From day 28, the pH in the supply area of the control barn was in an acidic range, and in the supply area of the experimental barn the pH was in an alkaline range. In addition, litter quality assessment revealed cake formation in the supply area of the control barn from day 14.

A decrease in litter DM increases the NH_3_ volatilization [42]. Groot Koerkamp [40] reported that 40–60% litter DM provides the best conditions for microbial growth and thus for the release of NH_3_. Therefore, Miles et al. [43] stated that 54–55% of the total NH_3_ generated by the litter can be expected near the water lines promoted by the low litter DM level. The dissociation equilibrium between ammonium (NH_4_^+^) and NH_3_ is strongly pH-dependent. With an increase in the litter pH above pH 7, N is increasingly released as NH_3_ due to the microbial implementation processes [44,45,46,47]. This is supported by a function set up by Kirchmann and Lundvall [48], explaining 79% of the variance in the extent of the NH_3_ volatilization from animal wastes due to an increase in pH value. Various studies on the storage of animal excrements showed that the NH_3_ release is promoted by aerobic conditions and inhibited by anaerobic conditions [49,50,51]. Anaerobic conditions during farmyard manure storage cause the degradation of organic material into volatile organic acids, resulting in a decrease in pH [52,53]. Under aerobic litter conditions, the volatile organic acids are degraded to CO_2_, which increases the pH with time [54]. From day 14, cake formation occurred in the supply area of the littered control barn. Cake formation is described as compacted litter at a height of 5 to 10 cm on the surface of the litter [55]. Chadwick [56] demonstrated an NH_3_ reducing effect of 50–90% by using compaction and covering cattle manure heaps. The opposite effect is shown during the turning of cattle manure, where the air exchange and the increase in the emitting surface area caused an increase in the NH_3_ concentration [57]. In the present study, loosening of litter also increased the NH_3_ concentration, as presented by the post-litter treatment NH_3_ concentration peak on day 11 of the winter fattening period.

In summary, the animals compacted the litter in the supply area of the littered control barn, resulting in cake formation from day 14. Cake formation led to anaerobic conditions, a decrease in litter pH, and thus an inhibition of the NH_3_ release. In contrast, animals were separated from their excrements by the elevated perforated floor in the supply area of the experimental barn. As a result, no compaction by the animals took place and aerobic conditions occurred. As mentioned above, aerobic conditions increased the pH to an alkaline range, which promoted the NH_3_ release and thus the NH_3_ concentration and NH_3_ ER.

Despite the higher NH_3_ concentrations and NH_3_ ER, the partially perforated flooring system promotes animal welfare as indicated by animal-based welfare indicators without affecting production performance [12]. Such conflicting goals between animal welfare vs. environmental aspects are already known in livestock husbandry [58]. For example, free-range and organic systems in broiler production are often associated with longer fattening periods in combination with higher feed consumptions and nutrient excretions. These systems promote animal welfare but have a negative impact on the environment [59,60].

Compared with previous systems, the partially perforated flooring system offers the possibility of implementing reduction measures that were not implementable before. Solution approaches can be used in the supply area, where more than 50% of the total NH_3_ generation is expected [43]. For example, underfloor air extraction has become established in pig production to eliminate released NH_3_ directly at the source [61,62]. Such a system could also be tested in combination with the partially perforated flooring system. Almeida et al. [31] mentioned a decrease in NH_3_ concentration of a totally (100%) perforated flooring system in combination with regular manure removal during the fattening period. It is known from aviary systems in laying hens that regular manure removal with manure belts reduces NH_3_ concentrations [40,63,64]. Manure belts underneath the elevated partially perforated area could be tested for regular manure removal in the supply area. The system could also be used for catching animals for slaughter and final manure removal of the whole barn. In addition, the use of acids in pig and cattle slurry has proven to be effective in reducing the pH and thus the NH_3_ release [65,66]. Similar approaches exist with pH-lowering litter additives in poultry production [67,68]. The partially perforated flooring system offers the possibility of testing a system to acidify the material below the elevated perforated area and thus reduce NH_3_ volatilization. The advantage is that the animals are separated from the acidified material due to the elevated perforated area. With these measures, the increased NH_3_ volatilization underneath the perforated area could be compensated for and largely avoided. Further research approaches are necessary to reduce the NH_3_ concentrations inside the barn as well as the resulting NH_3_ ER, contributing equally to animal welfare and environmental protection.

## 5. Conclusions

This study examined how a partially perforated (50%) flooring system affects NH_3_ emissions compared with a littered system in broiler housing. Compared with the littered flooring system, the material underneath the elevated perforated area was not compacted during the fattening periods. Therefore, aerobic conditions were present, which increased the litter pH and thus the NH_3_ concentration and NH_3_ ER in the final phase of the fattening period. Nevertheless, with the exception of the mechanical litter treatment in the winter fattening period, NH_3_ concentrations in the exhaust air were below 20 ppm for both floor types. Underfloor air extraction, manure belts, or acidification systems below the elevated perforated area are potential solution approaches to inhibit NH_3_ release that were not feasible before. Previous studies showed a positive effect of the partially (50%) perforated flooring system on health- and behavior-based welfare indicators without affecting production performance. In summary, with optimization measures, the partially perforated flooring system could contribute to an improvement in animal welfare and environmental protection at the same time. Further studies should be carried out with a focus on the partially perforated flooring system combined with NH_3_ reduction strategies from agricultural practice.

## Data Availability

The data presented in this study are available on request from the corresponding author.
